# 999 Developing and Implementing a Burn Care Curriculum for Physical and Occupational Therapy Clinical Education Students

**DOI:** 10.1093/jbcr/iraf019.530

**Published:** 2025-04-01

**Authors:** Deborah Knight, Courtney Comstock-Elizondo

**Affiliations:** Joseph M. Still Burn Center at Doctors Hospital; Joseph M. Still Burn Center at Doctors Hospital

## Abstract

**Introduction:**

The COVID pandemic had a large impact on our ability to recruit and retain burn therapists. Simultaneously, clinical placements in the acute care setting were limited. In PT and OT education programs, it is standard for students to receive a lecture on burns, yet it is a highly specialized domain of practice, requiring years of experience to achieve expertise. Clinical Education (CE) can be maximized as specialized training, utilized for recruitment and network development.

**Methods:**

In 2023, a Clinical Coordinator was selected from among the burn therapists and a formal CE curriculum was developed based on the Commission on Accreditation in PT Education (CAPTE) and the Accreditation Council for OT Education (ACOTE) standards for clinical placements. Using the APTA Acute Care Competencies, the AOTA Position Statement on Critical Care OT Practice Across the Lifespan, along with the Burn Therapist Competency Tool, Version 2, this curriculum was designed to introduce and incorporate entry-level burn-specific skills into the PT and OT students’ clinical learning experience and an interview process was initiated. From October 2023 to September 2024, 8 students (4 PT, 4 OT) successfully completed clinical placements at our center with five different Clinical Instructors (CI), utilizing this new curriculum. In addition to the curriculum, informal check-ins were provided for both students and their respective CI at mid-term and final assessments. Students provided a written assessment of the clinical rotation (CR) and placement site, as well as feedback on their experience with their CI. Written and verbal feedback was assessed and implemented as a quality improvement measure.

**Results:**

All 8 students successfully passed their CR. Job offers following completion of the CR increased by 61% compared to the prior four years. The direct hiring rate of students increased by over 250%, with 3 students referred to other burn centers for the first time. Overall student feedback was positive regarding their learning experience and all 8 students were able to provide constructive feedback which was then implemented by all repeat CIs.

**Conclusions:**

The development and implementation of a formalized CE curriculum for PT and OT students within the acute burn care context demonstrates positive outcomes and serves as a tool to facilitate consistent instruction while preparing for a career in burn care after graduation. CIs also benefited from an organized framework, were able to improve their mentorship skills, and strengthen constructive feedback for clinical students.

**Applicability of Research to Practice:**

There are no current standards of clinical education for PT and OT students related to the Acute Burn Care context. By establishing and assessing the success of a formalized Burn Rehabilitation CE curriculum, Burn Rehabilitation Practitioners can be identified and trained prior to entry into clinical practice.

**Funding for the Study:**

N/A

